# Effect of Gender, Physical Activity and Stress-Related Hormones on Adolescent’s Academic Achievements

**DOI:** 10.3390/ijerph17114143

**Published:** 2020-06-10

**Authors:** Ahmad H. Alghadir, Sami A. Gabr, Zaheen A. Iqbal

**Affiliations:** 1 Rehabilitation Research Chair, College of Applied Medical Sciences, King Saud University, Riyadh 11433, Saudi Arabia; aha@ksu.edu.sa (A.H.A.); dr.samigabr@gmail.com (S.A.G.); 2 Department of Anatomy, Faculty of Medicine, Mansoura University, Mansoura 35516, Egypt

**Keywords:** stress hormones, cortisol, serotonin, physical activity, adolescents, academic

## Abstract

Background: Physical activity (PA) has been shown to develop better fitness and body function in children. Various studies have shown that as the age of students increases, its correlation with school achievement decreases. Different hormonal changes during adolescence make it difficult to adjust in his/her environment, causing stress. To the best of our knowledge, no study has studied the correlation between stress-related hormones and school performance among adolescents. This study was conducted to evaluate physical activity and stress-related hormones, cortisol and serotonin, among school adolescents aged 12–18 years old and find their association with academic achievements. Methods: A total of 300 students were invited to participate in this study. Physical activity of the participants was assessed in relation to the time spent performing various physical activities. End of the academic year grades were obtained from the school as a collective measure of academic achievement and executive function. The levels of cortisol and serotonin were measured using the competitive immunoassay techniques. Results: There was a significant correlation between age, gender, BMI, cortisol, serotonin, physical activity score; and academic achievement, and executive functioning among participants. Academic achievement and executive functioning scores correlated positively with gender, serotonin, physical activity score, but negatively with age, BMI and salivary cortisol. Stepwise regression analysis showed that physical activity and demographic parameters and stress-related hormones, cortisol and serotonin, explained around 61.9–77.9% of academic performance and executive functioning variation in school adolescents, especially females. Conclusions: Optimal physical activity and release of stress-related hormones could be the determining factor for performance in school and other activities. These results should be taken into consideration while planning the school curriculum.

## 1. Introduction

Children with regular physical activity (PA) have been shown to have a better fitness and body function [1]. However, PA is often not promoted in schools due to thinking that it has a negative effect on academic grades [2]. With the recent advances in mobiles, television (TV) and internet, after school PA has also decreased [3,4,5]. To overcome this, various researchers have been trying to find a positive correlation between PA and school performance [6]. Although several reviews have reported positive association between PA and mental health, their weak methodologies have also been pointed out [7,8,9].

There are various studies that explore the effect of age and gender on school performance, which show that as the age of students increases, its correlation with school achievement decreases [10,11,12]. Gender has been reported to play an important part in academic achievement in school [13]. Although most studies report that girls outperform boys in school [14], some studies show that male students perform better in physical sciences and engineering while female students perform better in life sciences and languages [15,16]. However, the reason behind such differences in school performance based on gender is not much reported.

Previous studies have shown that PA is positively associated with cognitive performance and academic achievement among adolescents [17]. Following exercise training programs, positive effects on brain structures [18], cognitive performances [19] as well as academic achievements [9] have also been reported among adolescents in different studies [17,20,21]. These potential effects of PA are briefly based on the physiological changes that occur in adrenal function and need to be further explored.

Adolescents face various puberty-related changes in their bodies which makes their adjustment in social environments challenging [22]. In most cases, these changes affect their behavior and mode of lifestyle negatively, and further lead them to depression or perceived stress [23,24]. Various studies [25,26,27] have pointed out that such stressful life events have a negative effect on their physical and mental health, and play a potential role in high school dropout [25,26,27]. Greater changes in the levels of stress-induced hormones, cortisol and serotonin [28,29], have been reported in adolescents of both genders due to stressful life events. In addition, interaction between the cellular expression of serotonin transporter gene and stressful life events, which often causes depression, has also been reported [30,31].

Although some studies have reported an effect of programmed teaching approaches on stress response physiology and brain activity among children, there is little consensus about the nature of the association between stress hormones and academic achievements [29,31]. To the best of our knowledge, no study has correlated levels of stress-related hormones with school performance among adolescents. This study was conducted to evaluate physical activity and stress-related hormones among school going adolescents aged 12–18 years and find their association with academic achievements.

## 2. Materials and Methods

### 2.1. Participants

A total of 300 students of grades 7 and 9 from three different senior secondary schools with similar sociocultural environments and following a common prescribed syllabus and examination evaluation pattern were invited to participate in this study during the period of October 2018 to March 2019. Students with any acute or chronic health problems like diabetes, cardiovascular diseases, infections, asthma, malabsorption, physical disability, musculoskeletal disorders, mental disabilities, and/or concentration problems were excluded. All the parents and students were informed about the purpose and nature of this study, and their written informed consent was obtained in order to be included in the study. Ethical approval in compliance with the Helsinki Declaration was obtained from institutional research review board.

**Figure 1:**
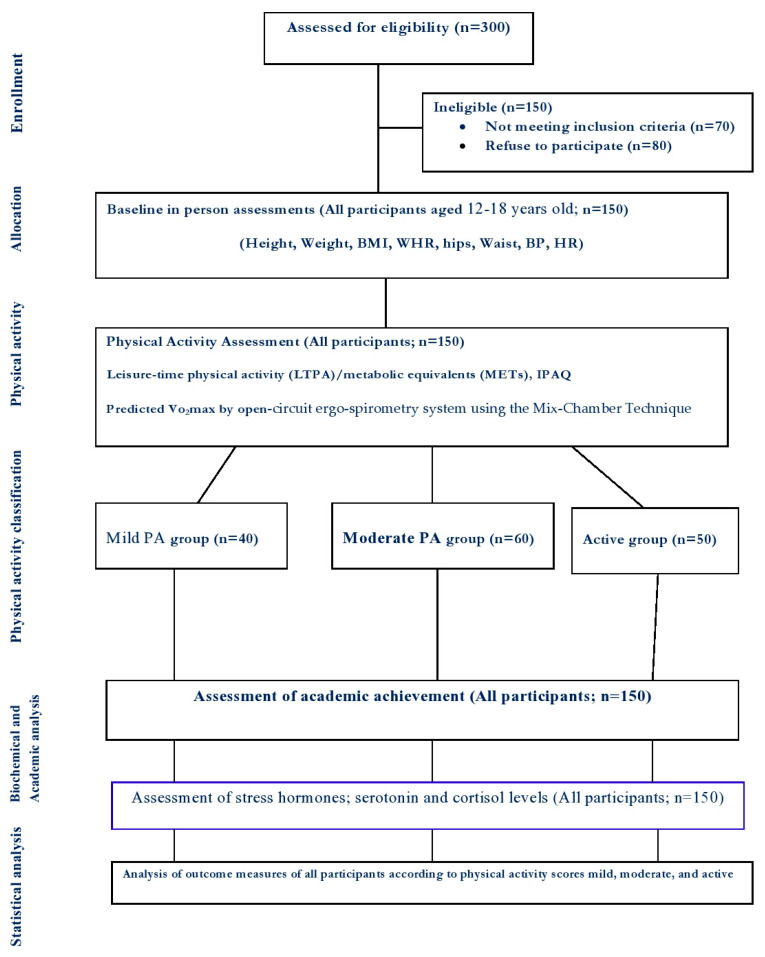
Outline of physical activity screening as well as associated assessments, LTPA: Leisure-time physical activity, METs: metabolic equivalents, IPAQ: International physical activity questionnaires, VO2 max, stress hormones, and academic achievements in all participants (n = 150).

After considering the inclusion and exclusion criteria, 150 students were included in the study (Figure 1). The demographic characteristics of the participants are shown in Table 1. They were classified into 3 groups; mild (n = 40; 25 boys, 15 girls), moderate (n = 60; 45 boys, 15 girls) and active (n = 50; 30 boys, 20 girls), based on the physical activity (see below).

### 2.2. Anthropometric Measurements

The height and weight were measured for all participants by using a tape measure and calibrated Salter Electronic Scales (Digital Pearson Scale; ADAM Equipment Inc., Columbia, MD, USA) respectively, with a standardized procedure [32,33]. Body mass index (BMI) was calculated as a measure of obesity as the ratio of weight (kilograms) to squared height (meters) [32,33,34,35]. In addition, hip and waist circumference (WC) were also measured as previously reported [32,33]. The waist circumference (WC) was measured at the midpoint between the last rib and the iliac crest using a tape measure (Ohaus^®^ 8004-MA; Ohaus, Parsippany, NJ, USA). Hip circumference was measured using a measuring tape starting at one hip and wrapping the tape around the other hip, and back to the point where started. It was made sure that the tape was over the largest part of the buttocks [32,33,34,35]. Healthy referenced cut-points derived previously were used to classify our data depending on its large sample size, age specificity, and relatively generalized ethnicity [34,35].

### 2.3. Assessment of Physical Activity

PA of the participants was assessed in relation to the time spent in performing various physical activities. According to the previous reports, a participant’s leisure-time physical activity (LTPA) was measured as metabolic equivalents (METs) [36,37,38,39,40,41]. METs were calculated as MET-minutes/week of the intensity of physical activity through the interview for each participant according to a formula used in recent studies [38,39]. Body mass, height, age, sex, and physical activity were used to calculate their basal metabolic rate (BMR) and total daily energy expenditure (TDEE) according to the Harris and Benedict equation [40,41]. In order to find the association between physical activity, stress-related hormones and academic achievements, participants were divided into 3 groups depending on the physical activity level [42]. They were classified into mild (≤500 METs-min/week), moderate (500–2500 METs-min/week), and active (≥2500 METs-min/week) groups.

- BMR calculation for men (metric)
  {BMR = 66.47 + (13.75 × weight in kg) + (5.003 × height in cm) − (6.755 × age in years)}
- BMR calculation for women (metric)
  {BMR = 655.1 + (9.563 × weight in kg) + (1.850 × height in cm) − (4.676 × age in years)}

### 2.4. Assessment of Maximum Aerobic Power (VO_2_ Max)

Although leisure-time physical activity (LTPA) was measured in metabolic equivalents (METs), it was validated and recommended by various research studies [36,37,38,39,40,41]. The maximum aerobic power (VO_2_ max) was measured to support the physical activity (LTPA), whereas physiological changes in the values of VO_2_ max and the responses to exercise activity based upon VO_2_ max was evaluated among younger ages [43].

Prior to the beginning of the test, the measurement of blood pressure was performed to get the participant’s baseline cardiovascular status, response to exercise/activity, and guide exercise prescription or gauging exercise capacity as previously reported [44,45].

Then, the individuals performed three minutes of warm up at the 3.1 km/h velocity. Heart rate (HR) was monitored in the electrocardiogram. The respiratory parameters were measured in an open-circuit ergo-spirometry system using the Mix-Chamber Technique [46,47,48].

At the active phase, the maximum aerobic power (VO_2_ max) for each participant was evaluated in the ergo-spirometry on treadmills (inclination 1%). It was at an initial velocity of 4.5 km/h, which increased gradually at 0.5 km/min. This increase in velocity remained until voluntary exhaustion or when one of the following criteria was reached: increase in the VO_2_ lower than 2 mL·kg^−1^ for the increase in the exercise intensity (plateau); expiratory exchange ratio higher than 1.1; maximum heart rate expected for the age was reached, calculated by the following formula (220-age) [46,47,48];

{[VO_2_ max (mL/kg·min) = VO_2_ {220-age − 73-(sex × 10)/HR-73-(sex × 10)]}

{[Vo_2_ (mL/kg/min) = (1.8 * work heart rate)/body weight]}

Sex = 0 for girls and 1 for boys; HR = Heart rate at final stage.

At the last phase, all participants go into resting stage or cool-down, which continued for 10 to 15 min, where the workload decreased gradually until the participant’s HR and blood pressure returned nearly to resting level. Throughout the entire training session, the heart rate of the participant was monitored using a portable heart rate monitor to maintain exercise intensity within the pre-calculated training heart rate [49].

### 2.5. Assessment of Respiratory Exchange Ratio (RER)

The amounts of oxygen consumed and carbon dioxide produced are measured in inspired and expired air, using an oxygen/carbon dioxide gas analyzer connected to a spirometer, as previously reported [50,51]. The measured concentrations and volumes of oxygen and carbon dioxide are brought together in a series of equations; whereas, the volume of oxygen per minute, known as VO_2_, and the volume of carbon dioxide produced per minute, known as VCO_2_ are determined [52,53].

The respiratory exchange ratio (RER) was calculated as the ratio of VCO_2_/VO_2_, which was significantly used to determine the proportion of carbohydrates and fats utilized, the energy expended per liter of oxygen consumed during resting or an activity stage [51,52,53]. Calculation of RER is commonly referred to utilization of energy from either carbohydrates or fats or both of them [53,54], whereas RER value < 0.71 refers to the occurrence of lipid oxidation, RER value > 1.0 refers to the occurrence of carbohydrate oxidation, and RER value between 0.71 and 1.0 refers as an index of fat to carbohydrate oxidation.

### 2.6. Assessment of Academic Achievement

At the end of the academic year, participants’ grades were obtained from the school. All the three schools followed the same prescribed syllabus and examination evaluation pattern. The mean grades in biology, chemistry, physics, English, French, mathematics, social sciences, history, geography, religion, physical education and health sciences were collectively reported as measures of academic achievement. Their grades ranged from 1.0, very bad to 10, outstanding. The performance in mathematics was analyzed separately as a measure of executive functioning [55].

### 2.7. Assessment of Serotonin and Cortisol Levels

The stress hormones were analyzed 2–3 months before examinations to avoid any interfering factors like anxiety. Cortisol level (pg/mL) was measured in the saliva samples of participants using the immunoassay technique according to the instructions of the competitive ELISA-kit (Diagnostics Biochem, Ontario, Canada, Inc.). Serotonin levels (ng/mL) were estimated in serum samples using the competitive immunoassay ELISA kit (KA1894, Novus Biologicals, Ontario, Canada, Inc.).

### 2.8. Sample Power Calculation

In this study, the G*Power software (Version 3.1.9.2.) was used to estimate the statistical power analyses of cofounders [56]. To observe statistically significant differences at α level of 0.05 at the power of 0.96 between the studied groups, a total sample size of 150 was needed. Thus, a sample size of 150 participants were at least sufficient to estimate the levels of adrenal stress hormones, serotonin and cortisol, along with the scores of academic achievements of the students with a precision of 10% and a power of 96%.

### 2.9. Statistical Analysis

All statistical analyses were achieved using the SPSS statistical software version 15.0 (SPSS Inc., Chicago, IL, USA). For the study sample, all studied variables were presented as means, standard deviations, or relative frequencies (n, %) respectively. Two-way analysis of variance, Mann–Whitney U test, and *t*-test parameters were performed to explore the differences in the levels of adrenal stress hormones and the scores of academic achievements in adolescents based on gender and PA scores.

Pearson’s correlation coefficients were measured to examine the relationships between serotonin and cortisol as markers of adrenal stress and the scores of academic achievements based upon PA outcomes and gender of the participants. The *p*-values < 0.05 were considered to be significant.

In addition, stepwise linear regression models with a multicollinearity test with a higher variance inflation factor (VIF ≤ 5) were used to estimate the correlation between the studied variables and academic achievements in the school for the adolescents. Only variables with odds ratios of at least 10% proportional increase and had the highest T-value with lower VIF ranges with strong significance were considered reliable confounders of a constant model. In this model, age, gender, BMI, cortisol, serotonin, and physical activity score (PA) were reliable independent confounders with lower VIF values and higher T-statistics with significance values. The confounders were step wisely estimated according to the following equations:

{(Y = β_0_ ₊ β_1_ (Age) ₊ β_2_ (gender) ₊ β_3_ (BMI) ₊ β_4_ (cortisol) ₊ β_5_ (serotonin)⁺ β_6_ (PA))⁺ έ)}

{(Multicollinearity variance inflation factor (VIF) = 1/1-R^2^)}

where Y refers to dependent variables, academic achievements (AA) and mathematics performance (MP).

## 3. Results

A total of 150 students aged 12–18 years (boys, n = 90; 60%) participated in this study. There was no significant difference between boys and girls participants in relation to adiposity parameters; BMI, WHR, WC, hips circumference. Clinical results of both genders showed normal blood pressures, maximum pulse averages, and glycated hemoglobin (HbA1c). At the resting stage, the levels of oxygen consumption (VO_2_), the released carbon dioxide (VCO_2_), as well as respiratory exchange ratio (RER), and maximum aerobic power (VO_2_max) were shown to be normal in both boys and girls participants (Table 1).

The participants who were physically less active (mild PA; ≤500 METs-min/week) were approximately 27% (n = 40) of the total participants. The remaining 63% were classified into moderate PA (n = 60; 40%) who had an LTPA-PA score of 500–2500 METs-min/week, and physically active students (n = 50; 33%) with an LTPA-PA score of more than ≥2500 METs-min/week (Table 2).

### 3.1. Comparison of LTPA, BMR, TEE, and VO_2_ Max Based on PA and Gender

Compared to participants with mild PA, LTPA, BMR, TEE, and VO_2_ max as parameters of PA scores were significantly (*p* < 0.001) higher in participants with moderate and active PA. Regarding gender differences, physically active girls showed significantly (*p* < 0.01) higher values of LTPA, TEE, and VO_2_ max compared to boys of the same respective group (Table 2).

### 3.2. Comparison of Stress-Related Hormones Based on PA and Gender

The participants with moderate to active PA showed a significant (*p* < 0.001) reduction in the levels of salivary cortisol and a release in the levels of serum serotonin compared to subjects with lower or mild PA, who represent abnormal levels of adrenal hormones (Table 2). Compared to boys of the same group, girls with active (*p* < 0.01) and moderate (*p* < 0.01) PA scores showed a higher level of serum serotonin and lower salivary cortisol (Table 2).

### 3.3. Comparison of Academic Achievements Based on PA and Gender

Regarding academic achievement, it was shown to be associated with the physical activity of the participants (Table 3). Lower scores of academic achievement were estimated at 27% of the students. In this physically less active group (mild PA), the academic scores were 4.68 ± 0.71 for boys and 4.96 ± 0.77 for girls, respectively. In addition, mathematics performance measured by executive function was 4.74 ± 0.68 for boys and 4.3 ± 0.65 for girls (*p* < 0.01), respectively, as shown in Table 3.

The results also showed that 63% of the students had higher scores of academic achievement (*p* < 0.001), as shown in Table 3. The data were significantly associated with physical activity scores. The results of academic achievements were significantly higher among students with moderate (*p* < 0.001) and active (*p* < 0.001) PA scores compared to those with mild PA scores (Table 3). Gender differences also showed that girls with moderate (*p* < 0.001) and active group (*p* < 0.001) PA scores showed a significantly higher academic and executive functioning score compared to boys of the same respective group (Table 3).

### 3.4. Association between Gender, Age, VO_2_ Max, BMR, TDEE, LTPA, BMI, Stress-Related Hormones, PA Scores, and Academic Achievement

Stepwise regression analysis and a multicollinearity test with higher variance inflation factor (VIF ≤ 5) were performed to estimate the reliable independent confounders, which could interfere or predict the status of academic and mathematic performance measured by executive function among adolescence students. Gender, age, BMI, stress hormones (cortisol and serotonin), and PA scores were included in the stepwise linear regression models, as they showed higher T-values and lower values of VIF as a measure of a multicollinearity with strong significance (*p* ≥ 0.05), as shown in Table 4. On the other hand, VO_2_ max, BMR (kcal/day), TDEE (kcal/day), LTPA (METs-min/week), waist, hips, WHR, and mean HbA1c values showed lower T-values with higher VIF scores, hence, deleted from the proposed model (Table 4).

The data showed that the selected independent confounders—age, gender, BMI, cortisol, serotonin, and PA score—were significantly associated with academic achievement and executive functioning among participants. Academic achievement and executive functioning scores correlated positively with gender, serotonin, PA score, but negatively with age, BMI and salivary cortisol (Table 5). In addition, the proposed stepwise regression model showed that PA, demographic parameters and stress-related hormones (cortisol and serotonin) explained around 61.9–77.9% of academic performance and executive functioning variation in school adolescents, especially females (Table 5).

## 4. Discussion

This study was conducted to evaluate physical activity and stress-related hormones, and their association with academic achievements among school adolescents aged 12–18 years. At least 63% of the participants were found to be physical active. Among them, PA was found to be positively associated with adrenal functions as measured by stress-related hormones and overall academic achievement and executive function.

The results of this study further show that academic achievement and executive functioning scores correlated positively with gender, serotonin, PA score, and negatively with age, BMI and salivary cortisol. Although there are various studies that report difference in school performance based on gender, learning difficulties and PA [57,58,59], to the best of our knowledge, no study has correlated the level of stress-related hormones with school performance among adolescent students. Studies in the available literature have mostly studied primary school children and reports that study such effects beyond this age level are limited.

Our results also show that level of salivary cortisol is lower and serum serotonin is higher in all participants in the moderate and active groups in comparison to the mild group. Although low levels of cortisol are desirable at rest, its increased level during mental engagement has been associated with higher levels of cognitive ability [60]. Such elevation has been shown to promote memory and learning [61]. Serotonin plays an important role in neural plasticity and development [62,63]. Studies have shown that adolescents who report to be happy and satisfied in the school environment are less likely to suffer from emotional and behavioral problems [64]. Academic competition in school can be the cause of stress, which can lead to depression and anxiety disorders [65]. It is primarily mediated by cortisol, which is released by hypothalamic–pituitary–adrenal axis (HPA) [66]. Furthermore, HPA functioning has been shown to be altered by exposure to stress, forming a vicious cycle [67].

Exercise, in any form induces physiological changes in the body that affects its neurobiological system [68], and prevents depression through increases in serum level of serotonin [69]. In our results, a higher serotonin level has been shown to be associated with better school performance in physically more active adolescents. This is in accordance with previous studies that showed that the serotonin transporter genotype and its higher expression rates are potentially associated with memory, attention, performance, and high IQ [70,71,72].

The academic performance of the students have been shown to be positively correlated with age, gender, vitamin E level, total antioxidant capacity, and physical activity [41]. PA has been shown to improve body fitness that has been shown to increase neural transmission activity in the brain [7]. It further increases attention and cognitive control for better academic achievement [57,73]. On the other hand, lower PA and sedentary lifestyles including more TV viewing have been associated with poor cognitive and executive functions at middle age [5]. Consistent with previous studies, our results also show that participants in the moderate and active PA groups have better school per performance in comparison to those in the mild group. Owing to the benefits of PA, minimum target PA has been proposed according to age, which should be achieved on a daily basis [74]. A child spends at least eight hours in school, which forms a significant part of the day. It can play a significant role to promote PA among them [75]. Various activities during lunch breaks and other periods in classroom, and physical education [76], have been proposed for this without affecting normal studies.

Studies have shown potential association between body compositions, sitting time spent while using computers and TV, and PA based on ethnicity and nationality [42,77]. Various studies that correlate PA with academic performance based on gender and have found mixed association. Some of them show positive effects in girls [78] while others in boys [79]. The findings of this study show that girls are better performers than boys in school among all the three PA groups. Although gender differences in academic performance have been reflected in studies conducted around the world [64,80], differences based on nationalities are apparent. Various developmental, cognitive, psychological, social and cultural factors can be the reason behind such differences [81,82,83]. This is the first of its kind study in the region where genders are highly segregated. Such differences should be kept in mind while framing the national curriculum for schools.

This study used self-reported data from participants and their parents that raise the possibilities of over or underestimation of their experiences. It has been reported that socioeconomic status and environment also affect school performance and body response under stress [75,76,77,78,79,84,85]. Thus, these factors should be considered in future studies regarding PA, gender and stress-related hormones and academic performance.

## 5. Conclusions

Academic achievement and executive functioning scores correlated positively with gender, serotonin, PA score, but negatively with age, BMI and salivary cortisol in adolescent students. Optimal PA and release of stress-related hormones could be the determining factor for performance in school and other activities. These results should be taken into consideration while planning the school curriculum.

## Figures and Tables

**Table 1:** General characteristics of participants according to gender (n = 150).

Parameters	Boys	Girls	Total
	(n = 90, 60%)	(n = 60; 40%)	(n = 150)
Age (years)	16.5 ± 0.76	16.11 ± 0.8	16.3 ± 0.78
BMI (kg/m^2^)	23.8 ± 1.47	22.46 ± 1.18	23.13 ± 1.32
Waist (cm)	86.6 ± 8.34	84.4 ± 9.3	85.5 ± 8.82
Hips (cm)	88.12 ± 9.17	88.7 ± 10.4	8.41 ± 9.8
WHR	1.0 ± 0.11	0.97 ± 0.14	0.98 ± 0.12
MP (beat·min^−1^)	7.5 ± 10.7	81.6 ± 10.8	79.5 ± 12.0
Systolic BP (mmHg)	102 ± 1.3	106 ± 2.7	104 ± 2.0
Diastolic BP (mmHg)	89.2 ± 2.86	84.7 ± 4.5	86.95 ± 3.7
Mean HbA1c value, %(SD)	2.98 ± 0.41	3.4 ± 0.85	3.19 ± 0.63
VO_2_ (mL/min)	1769 ± 237	1778 ± 239	1780 ± 243
VCO_2_ (mL/min)	1582 ± 214	1579 ± 212	1586 ± 215
RER (VCO_2_/VO_2_)	0.87 ± 0.05	0.86 ± 0.06	0.89 ± 0.06
VO_2_ max (ml·kg·min^−1^)	42.8 ± 3.7	43.4 ± 4.3	42.9 ± 4.1

Values are expressed as mean ± SD; WHR: waist to hip ratio; BMI: body mass index; VO_2_: oxygen consumption; VCO_2_: carbon dioxide production per minute; RER: respiratory exchange ratio; MP: maximum pulse averages.

**Table 2:** Associations of cortisol, serotonin, BMR, TDEE, and VO_2_ max with physical activity among participants (n = 150).

Parameters	Mild (n = 40; 27%) (≤500 METs-min/week)		Moderate (n = 60; 40%) (500–2500 METs-min/week)		Active (n = 50; 33%) (≥2500 METs-min/week)	
	Boys	Girls	Boys	Girls	Boys	Girls
	(n = 25)	(n = 15)	(n = 45) ^a^	(n = 15) ^b^	(n = 30) ^b,c^	(n = 20) ^b,c^
LTPA (MET-H/week)	61.52 ± 7.2	65.1 ± 9.7	104 ± 11.4	100 ± 10.4	157.13 ± 10.1	155.2 ± 10.5
BMR (kcal/day)	1.99 ± 0.44	1.8 ± 0.48	3.42 ± 0.62	3.56 ± 0.8	4.13 ± 0.57	3.7 ± 0.58
TDEE (kcal/day)	2.2 ± 0.65	1.82 ± 0.41	3.47 ± 0.8	3.16 ± 0.73	4.36 ± 0.67	4.63 ± 0.87
VO_2_ max (mL/kg*min)	29.5 ± 2.7	31.7 ± 3.8	37.8 ± 3.4	36.9 ± 6.1	42.5 ± 3.5	45.3 ± 2.1
Cortisol (pg/mL)	58.6 ± 6.9	60.3 ± 7.3	42.1 ± 7.9	36.1 ± 5.9	29.1 ± 8.0	23.6 ± 6.9
Serotonin (ng/mL)	38.44 ± 9.8	33.6 ± 1.1	46.8 ± 8.3	39.8 ± 9.8	59.25 ± 6.9	48.6 ± 6.4

Values are expressed as mean ± SD; ^a^ *p* < 0.05, ^b^ *p* < 0.01 (boys vs. girls), ^c^ *p* < 0.001 (moderate and active versus mild PA). BMR: basal metabolic rate; TEE: total energy expenditure; TAC: total antioxidant capacity.

**Table 3:** Associations of academic achievement and executive function with gender and physical activity among participants (n = 150).

Parameters	Mild (n = 40; 27%) (≤500 METs-min/week)		Moderate (n = 60; 40%) (500–2500 METs-min/week)		Active (n = 50; 33%) (≥2500 METs-min/week)	
	Boys	Girls	Boys	Girls	Boys	Girls
	(n = 25)	(n = 15)	(n = 45) ^b,c^	(n = 15) ^b,c^	(n = 30) ^b,c^	(n = 20) ^b,c^
Academic Achievement	4.68 ± 0.71	4.96 ± 0.77	6.2 ± 0.34	7.2 ± 0.77	7.3 ± 0.29	7.6 ± 0.36
Executive function	4.74 ± 0.68	4.3 ± 0.65	6.3 ± 0.26	6.86 ± 0.66	6.9 ± 0.43	6.98 ± 0.74

Values are expressed as mean ±SD; ^a^ *p* < 0.05, ^b^ *p* < 0.01 (boys vs. girls) in respective group, ^c^ *p* < 0.001 (moderate and active PA versus mild PA).

**Table 4:** Multicollinearity test diagnostics and selection of stepwise linear regressed cofounders.

Parameters	AA Score				MP Score			
	R-Squared	T-Value	VIF	*p*-Value	R-Squared	T-Value	VIF	*p*-Value
Mean HbA1c value	0.984	−1.9752	11.45	0.245	0.86	−1.674	12.86	0.130
VO_2_ max	0.962	−1.9894	14.56	0.176	0.961	−1.328	13.96	0.147
LTPA (MET-H/week)	0.94	1.612	12.4	0.157	0.974	1.354	13.23	0.125
BMR (kcal/day)	0.987	−1.389	11.8	0.258	0.981	−1.769	9.15	0.231
TDEE (kcal/day)	0.941	0.9781	8.63	0.215	0.897	0.239	10.3	0.124
Waist (cm)	0.86	1.421	16.73	0.145	0.974	1.974	15.98	0.324
Hips (cm)	0.927	−1.356	13.49	0.113	0.869	−1.743	12.78	0.298
WHR	0.9716	0.456	12.31	0.178	0.897	0.9186	13.87	0.182
Age	0.34	4.783	1.45	0.003	0.48	3.451	2.95	0.002
Gender	0.56	5.375	4.2	0.002	0.78	2.789	3.789	0.001
BMI (kg/m^2^)	0.38	4.17	3.731	0.001	0.58	4.125	1.974	0.005
Cortisol (pg/mL)	0.64	7.56	3.1	0.004	0.672	6.315	2.561	0.001
Serotonin (ng/mL)	0.372	6.78	1.39	0.001	0.741	5.7821	3.891	0.002
Physical activity score	0.692	6.75	1.98	0.003	0.497	5.897	2.89	0.003

(VIF ≤ 5): higher variance inflation factor; AA; Academic Achievement; MP: mathematics performance; BMI: body mass index; WHR: waist to hip ratio. The correlation of the studied parameters assessed according to the following stepwise regression equations with Multicollinearity test; Y = ({β_0_ ⁺ β_1_ (Age) ⁺ β_2_ (gender) ⁺ β_3_ (BMI) ⁺β_4_ (cortisol) ⁺ β_5_ (serotonin) ⁺ β_6_ (PA) ⁺ έ)}; Multicollinearity variance inflation factor (VIF) = ({1/1-R^2^)}. Y (AA/MP); VIF (≥ 10).

**Table 5:** Results of stepwise linear regression analysis of academic achievement and mathematics performance predicted by cortisol, serotonin, BMI, and physical activity score among participants (n = 150).

Parameters	Academic Achievement	Mathematics Performance
	β (R^2^) ^a^	β (R^2^) ^b^
Age	25.8 (−0. 45)	15.4 (−0.41)
Gender	6.1 (0.041)	8.1 (0.028)
BMI (kg/m^2^)	24.5 (−0.071)	18.1 (−0.061)
Cortisol (pg/mL)	11.9 (−0.059)	12.5 (−0.063)
Serotonin (ng/mL)	9.3 (0.037)	7.5 (0.064)
Physical activity score	0.28 (0.036)	0.31 (0.089)
ΣR^2^ (%)	77.9	61.9

Notes: Beta coefficient (β) and cumulative R^2^ derived from the stepwise regression analysis model showed additional significant variables added to the model via bivariate analysis. ΣR^2^ = summation of cumulative values of R relating to studied variables, ^a^ *p* < 0.05; ^b^ *p* < 0.01. The correlation of the studied parameters assessed according to the following stepwise regression equations with Multicollinearity test; AA; Academic Achievement; MP: mathematics performance; Y = ({β_0_ ₊ β_1_ (Age) ₊ β_2_ (gender) ₊ β_3_ (BMI) ₊ β_4_ (cortisol) ₊ β_5_ (serotonin) ₊ β_6_ (PA) ₊ έ)}; Multicollinearity variance inflation factor (VIF) = ({1/1–R^2^)}. Y (AA/MP); VIF (≥ 10).

## Abbreviations

PA	Physical activity
TV	Television
LTPA	Leisure-time physical activity
METs	Metabolic equivalents
BMR	Basal metabolic rate
TDEE	Total daily energy expenditure
VO_2_max	Maximum aerobic power
HR	Heart rate
HPA	Hypothalamic-pituitary-adrenal axis
